# How a well-grounded minimal important difference can enhance transparency of labelling claims and improve interpretation of a patient reported outcome measure

**DOI:** 10.1186/1477-7525-4-69

**Published:** 2006-09-27

**Authors:** Jan L Brożek, Gordon H Guyatt, Holger J Schünemann

**Affiliations:** 1 Department of Medicine, Jagiellonian University School of Medicine, Krakow, Poland; 2 Polish Institute for Evidence Based Medicine, Krakow, Poland; 3 CLARITY Research Group, Department of Clinical Epidemiology and Biostatistics, McMaster University; Hamilton, Ontario, Canada; 4 Department of Medicine, McMaster University; Hamilton, Ontario, Canada; 5 Division of Clinical Research Development and Information Translation/INFORMA & CLARITY Research Group, Department of Epidemiology, Istituto Regina Elena/Italian National Cancer Institute, Via Elio Chianesi 53, 00144 Rome, Italy

## Abstract

The evaluation and use of patient reported outcome (PRO) measures requires detailed understanding of the meaning of the outcome of interest. The Food and Drug Administration (FDA) recently presented its draft guidance and view on the use of PRO measures as endpoints in clinical trials. One section of the guidance document specifically deals with advice about the use of the minimal important difference (MID) that we redefined as the smallest difference in score in the outcome of interest that informed patients or informed proxies perceive as important. The advice, however, is short, indeed much too short. We believe that expanding the section and making it more specific will benefit all stakeholders: patients, clinicians, other clinical decision makers, those designing trials and making claims, payers and the FDA.

There is no "gold standard" methodology of estimating the MID or achieving the meaningfulness of clinical trial results based on patient reported outcomes. There are many methods of estimating the MID usually grouped into two distinct categories: anchor-based methods, that examine the relationship between scores on the target instrument and some independent measure, and distribution-based methods resorting to the statistical characteristics of the obtained scores.

Estimation of an MID and interpretation of clinical trial results that present patient important outcomes is demanding but vital for informing the decision to recommend approve a given intervention. Investigators are encouraged to use reliable and valid methods to achieve meaningfulness of their results, preferably those that rely on patients to estimate what constitutes a minimal important, small, moderate, or large difference. However, acquiring the meaningfulness of PRO measures transcends beyond a concept of the MID and we advocate that dichotomizing the scores of patient-reported outcome measures facilitate interpretability of clinical trial results for those who need to understand trial results after a labelling claim has been granted. Irrespective of the strategy investigators use to estimate these values, from the individual patient perspective it is much more relevant if investigators report both the estimated thresholds and the proportion of patients achieving that benefit.

## Background

The Food and Drug Administration (FDA) presents its draft guidance and its view on the use of patient-reported outcome (PRO) measures as endpoints in clinical trials in this issue of Health and Quality of Life Outcomes [1]. It includes information on how sponsors could use study results based on these measures to support claims in their product labelling that carries important implications for study design and interpretation of the findings [1]. The advice, however, is short, indeed we believe much too short.

The evaluation and use of PRO measures requires detailed understanding of the meaning of the outcome of interest. Achieving this understanding presents a considerable challenge even for seemingly straightforward dichotomous outcomes such as stroke, myocardial infarction, or death [2,3]. The complexity increases with the realization that no binary outcome is truly unambiguous: deaths can be painful or painless, strokes can be mild or severe, and myocardial infarctions can be large and complicated or small and uncomplicated. The way the investigators present the results of clinical trials also influences clinicians' willingness to undertake a specific action [4-7]. This problem becomes even more complex when one considers that different patients may place a different value on a particular benefit (inter-individual variation) or even the same patient may place a different value on the same benefit (intra-individual variation), depending on the circumstances. These difficulties occur despite the ease with which one can generally communicate an event such as stroke or death.

The challenges increase further, when one faces PRO scores expressed in unfamiliar ordinal or continuous scales. Even those familiar with the concept of PRO or health related quality of life (HRQL) assessment generally have no intuitive notion of the significance of a change in score of a particular magnitude on most instruments that investigators use to measure the severity of outcomes such as stroke or myocardial infarction.

Therefore, one can frame the problem as an issue of interpretability: what changes in score correspond to trivial, small, moderate, or large patient benefit or harm [8].

The FDA guidance dedicates section IV.C.4 (Choice of Methods for Interpretation) to this particular issue and describes "some of the methods that have helped sponsors and the FDA interpret clinical trial results based on PRO endpoints". We believe that expanding this section and making it more specific will benefit all stakeholders: patients, clinicians, other clinical decision makers, those designing trials and making claims, payers and the FDA.

## Discussion

The Authors of the guidance focused their attention on the minimal important difference (MID). Therefore, we will centre our attention on 4 questions related to the MID: 1) what is the MID; 2) why is the MID important; 3) how to estimate the MID; and 4) when the MID is the proper approach to claiming efficacy and how can it be used by clinicians to understand claims based on clinical trials using PRO measures. Acquiring the meaningfulness of PRO measures transcends beyond a concept of the MID, so we will supplement the discussion about questions 3 and 4 with a consideration on other related approaches investigators used to achieve interpretability of PRO instruments.

To place the interpretation of PRO measure scores in context, before we address the specific issues, we suggest, that the reader conceptualizes these scores as any continuous (e.g. visual analogue pain scale, height) or discrete, in particular ordinal (e.g. stage 1 through stage 4 cancer, severity of pain: none, mild, moderate, severe) variable. It may also be helpful to visualize the PRO score as a surrogate outcome measure that needs some "mapping" into another meaningful, patient-important outcome in order to gain interpretability.

### What is the minimal important difference?

#### Definition

The FDA document does not provide a *sensu stricto *definition of the MID obtained with a PRO measure and confines itself to the notion of "meaningful change" or "effect that might be considered important" [1]. We suggested that the MID be the smallest difference in score in the outcome of interest that informed patients or informed proxies perceive as important, either beneficial or harmful, and that would lead the patient or clinician to consider a change in the management [9,10].

We place a greater weight on the preferences of informed patients than of informed proxies (including clinicians) in determining the MID. We should consider the MID estimates of informed proxies only if informed patients cannot make decisions about the management of their disease or if patients prefer informed proxies, including clinicians, to make these decisions. It is also important to bear in mind that any change in management will depend on the associated downsides, including harm, cost and inconvenience.

#### Implications

This definition of the MID precludes making its estimates for outcomes that are remote from those important, in themselves, to patients, such as spirometry or laboratory exercise capacity. It also suggests that only if one had reason to question the reliability or accuracy of data from patients would one rely on proxies to provide estimates of the MID. If one accepts that PRO measurement must be fundamentally patient-important, the first choice for establishing the MID should be a patient-based approach. Relative to patients, clinicians may overemphasize treatment effects [11] and agreement between patients and proxies in rating the PROs is far from perfect [12-15]. To be maximally informative, representative samples of informed patients or if necessary their proxies should provide estimates of the MID.

### Why do we need a MID?

There are several reasons for which the concept of MID seems useful and investigators should derive it from patients. First, it appears easily understood by clinicians and investigators as a key concept when one considers the problem of interpretability of PRO scores. Second, it emphasizes the primacy of the patient's perspective and implicitly links that perspective to that of the interpretation by clinicians. Third, the choice of what constitutes a MID will inform judgments about the successfulness of an intervention. Fourth, it helps estimating the required sample size of clinical trials, and the very design of these studies. Fifth, an individual patient achieving the score equal or greater than the MID might be considered a beneficiary of the intervention, what would lead to the definition of a responder, as the authors of the guidance suggested. However, one should be cautious as it is certain that the MID varies across patients and possibly also across patient groups [16]. Since the MIDs are usually derived from the groups of patients, the description of responders based on the MID should be used with great care and with full disclosure to readers how it was obtained.

### How does one estimate the MID?

Unfortunately, there is no "gold standard" methodology of achieving the meaningfulness of PRO scores, estimating the MID, or interpreting these scores. This is part of the reason why interpreting PRO measures is indeed a demanding task. A possible and widely used technique would be to approach a group of experts and ask them whether the particular PRO score looks like a reasonable measure of what is important to patients, as they perceive it. This technique may be termed analogous to face validity. However, as described above this approach is based solely on the opinion of experts, and because the experts' perception of what is important to patients tends not to mirror what in fact it is [11,15], this method must he regarded as a weak means to establish a score that would represent the MID for patients. Fortunately, less medico-centric techniques are available, although none of them is perfect. The authors of the FDA guidance name four examples of derivation of the MID: "mapping changes in PRO scores to clinically relevant and important changes in non-PRO measures of treatment outcome", "mapping changes in PRO scores to other PRO scores", "using an empirical rule", and "using a distribution-based approach". We think the users of the guidance would benefit from some explanation added to this presentation by giving specific examples or descriptions.

#### Patient versus population perspective

An important issue in shaping the interpretability of a PRO score is whether one makes inferences about patient important change with respect to individuals or populations [17]. One frequently distinguishes between the significance of a particular change in score in an individual patient and a change of the same magnitude in the mean score of a group of patients [18]. From the point of view of the individual, a worthwhile change may be the one that results in a meaningful reduction in symptoms or improvement in function. In contrast, a change in mean score of a magnitude that would be trivial in an individual (e.g., 2 mm Hg reduction in blood pressure), may translate into a large number of benefiting patients in a population (e.g. reduced strokes).

There are two reasons for this difference in interpretation. First, one might consider particular change in score (e.g. 2 mm Hg in blood pressure) in an individual trivial, that is within the error of measurement. In this sense, the change is trivial because one does not believe it is real. On the contrary, relatively modest improvements at the individual level may be rated as important when considered at the group level. The second reason for the distinction between interpretation of individual and group differences is that not every individual in the population does experience the same change in outcome – even groups with negligible mean changes in PRO scores (or any outcome expressed as a mean score) are likely to contain individual patients whose improvement is noteworthy and leads to a reduced stroke risk [19]. From the group perspective individual variability is considered random variation associated with a measurement error. Therefore, from the individual patient perspective this very variability in individual response highlights the fundamental deficiency of summarizing treatment effects as a difference in means. Let us assume there is a threshold below which any change in status has no important consequences for the patient (i.e. the MID), and mean change in a group is below that threshold. If the distribution of change with treatment is narrow, it is possible that no patient will achieve an important benefit with treatment. On the other hand, if the distribution of change is large, it is likely that a substantial number of patients may achieve a benefit.

Inferences at the group or population level are likely to be informative with respect to the decisions regarding health care policy and inferences at the level of an individual are most relevant to the decisions concerning the management of individual patients.

Regardless of the chosen perspective, investigators have used two easily separable strategies to achieve an understanding of the meaning of scores of a given instrument [18]. The first relies on anchor-based methods and examines the relationship between scores on the target instrument (the instrument for which interpretation is in question) and some independent measure, termed an anchor. The FDA guidance refers to this strategy as "mapping". The second strategy is based on the statistical characteristics of the obtained PRO scores and is termed distribution-based. These later methods differ from anchor-based approaches in that they interpret results in terms of the relation between the magnitude of effect and some measure of variability in results.

For an in-depth review of these methods we refer the Readers to the work by Crosby [20] and Guyatt [17]. Herein we will present only the most general aspects of anchor- or distribution-based approaches.

#### Anchor-based approaches to estimating a meaningful change in PRO measure

Anchor-based methods compare PRO measures to an anchor that is itself interpretable, i.e. has a known relevance to patients. For example, a global rating of change [21-24], status on an important and easily understood measure of function [25], the presence of symptoms [26], mean scores of patients with a particular diagnosis [27-30], disease severity [31], response to treatment [31,32], or the prognosis of future events such as mortality [26,33,34], job loss [26,35,36] or health care utilization [37] can provide a useful anchor. Anchor-based methods require at least moderate correlation of the change on the anchor with the change on the target instrument.

One can subclassify anchor-based approaches into those that solve the interpretability problem in a single step – presenting population differences in status on multiple anchors – which one may call a population-focused approach, and those, that require two separate steps – first establishing the MID and then examining the proportion of patients who achieved the MID – which one may call an individual-focused approach.

The population-focused approach classifies patients in terms of the population to which they belong and is analogous to establishing construct validity, in that multiple anchors are generally required. In contrast, the individual patient-focused strategy tends to focus on a single anchor that is usually designed to establish a MID, but not necessarily so. This approach is analogous to criterion validity.

Those taking the individual patient-based approach usually attempt to identify a threshold between a change in score that is trivial and a change that is important (i.e. the MID). Those taking the population-based approach most commonly avoid identifying such a threshold, but offer relationships between target measure and multiple anchors instead, implicitly acknowledging that the threshold may vary, depending on the population under study and the range and severity of the problems being measured by the PRO instrument in question.

Having chosen a single-anchor approach, investigators may use alternative analytic strategies that will lead to different estimates of the MID [38]. The simplest and so far most widely used approach is to specify a result or a range of anchor instrument results that correspond to the MID and calculate the target score matching that value. The commonly used alternative is the use of receiver operating characteristic curves adopted from diagnostic testing [39-41]. This strategy classifies each patient according to the anchor instrument as experiencing an important change or not experiencing such a change. Investigators then test a series of cut-off points to determine the number of misclassifications. These misclassifications correspond to false-positive results (patients mistakenly categorized as changed) and false-negative results (patients mistakenly categorized as unchanged). The optimal cut-off point will minimize the number of misclassifications.

#### Distribution-based approaches to estimating a meaningful change in PRO score

Distribution-based methods interpret results in terms of the relation between the magnitude of effect and some measure of variability in results. Three categories of distribution-based approaches can be recognized [20]. The first approach depends on statistical significance and rates the score change in relation to the probability that this change is a result of a random variation of scores. Paired t-statistic [42] and growth curve analysis [43] are the examples. A second approach evaluates the score change in relation to sample variation: baseline standard deviation of patients [44,45], variation of change scores [24], and variation of change scores in a stable group [46]. The third approach evaluates the score change in relation to measurement precision. Examples include standard error of the mean [47] and a reliable change index [48]. As a measure of variability, investigators may choose between-patient variability (for example, the standard deviation of patients at baseline) or within-patient variability (for example, the standard deviation of change in the PRO that patients experienced during a study).

The most widely used distribution-based method is the between-person standard deviation, often referred to as effect size [44,45]. The group from which it is drawn is typically the control group at baseline or the pooled standard deviation of the experimental and control groups at baseline. Cohen [44] provided a rough rule of thumb to interpret the magnitude of the effect sizes. Changes in the range of 0.2 standard deviation units represent small changes, 0.5 – moderate changes, and 0.8 – large changes. Some recent empirical studies suggest that Cohen's guideline may in fact be generally applicable [49], but other authors propose that the MID is in the range of 0.2 to 0.5 standard deviation unit [50] or corresponds with an effect size of 0.5 [51,52].

The advantage of distribution-based methods is that the values are easy to generate in contrast with the work needed to generate an anchor-based interpretation. These methods have two basic limitations: estimates of variability differ from study to study and there is no intuitive meaning of the effect size (standard deviation units).

### How does the MID help to make sense of the results of clinical trials?

Describing the choice of the methods for interpretation of PRO instruments the authors of the FDA guidance addressed only the issue of deriving the MID leaving the issue of the very interpretation of clinical trial results based on these instruments unanswered. We have advocated that dichotomizing the results of a PRO measure facilitates interpretation of the clinical trial utilizing HRQL instruments [53,54]. Considering the above described approaches to achieve meaningfulness of PRO scores it is evident that one does not have to estimate the MID to grasp the meaning of particular scores.

#### Dichotomizing the distribution of scores

We have argued that one possibility is the use of intuitive thresholds to interpret PRO scores. To facilitate interpretability of clinical trial results, researchers can report thresholds that either refer to an absolute score (e.g. one can consider patients above a certain score as having achieved the outcome) or a change in score (e.g. one can consider patients' PRO measure as having improved or deteriorated if they achieve a certain change in score). For the absolute score, while interpreting the results of a trial, one could consider the proportion of patients who achieve a given mean score for which anchors exist before and after an intervention. For the change score approach, one could consider the proportion of patients who have changed by a certain score, for instance of 10. Researchers may report the results as a categorized distribution of the proportion of patients who achieved certain improvement in PRO measure. We also argued that using the example of the SF-36 instrument from the Medical Outcomes Study [55], the proportion of patients who are able, according to scores on the Physical Function scale (range 0–100), to walk a distance of one block (approximately 100 meters) without difficulty would be 32% for a score of 40, 50% for a score of 50, and 79% for a score of 60. Increasing the score from 40 to 50 indicates that 18% more people state that they can walk without serious limitations, and increasing it from 50 to 60 – that 29% more can walk one block, etc. From the group perspective, one could interpret a score of 50 as corresponding to approximately 50% of patients being able to walk one block. From an individual patient perspective, a score of 50 indicates a 50% chance that the patient is able to walk one block. If an intervention improved this score to 60, there would now be a 79% chance, or a 29% increase, of this patient's ability to walk one block. This interpretation is based on the assumption that the patient has similar characteristics to the population from whom these values are obtained.

#### Interpretation aids

Another example for the use of content-based interpretation of PRO measures is the construction of interpretation aids. Valderas et al. applied a specific model of item response theory (IRT) to an instrument measuring perceived visual function, the Visual Function Index (VF-14) [56]. This instrument asks respondents to rate the difficulties they have with their vision during performance of 14 everyday activities. Valderas et al. developed simple interpretation aids, that may facilitate the understanding of a particular score. The items were ordered according to their difficulty and used in the construction of a 'ruler' aid. This aid indicates the expected performance of an average patient with a given score. The authors have chosen a VF-14 score at which 50% of respondents have no difficulty performing a given task. For instance, a score of 97 indicates that 50% of respondents can drive without difficulty at night in regard to their visual function. A score of 75 indicates that 50% of respondents have no difficulty reading small print, 48 – watching TV and seeing steps, 36 – recognizing people when they are close, etc. Obviously, the authors could have chosen a score at which any other proportion of respondents has no difficulty performing a given task, but using a cut-off of 50% simplifies interpretation because it implies a 1 to 1 chance. This method of developing interpretation aids could be applied to many other PRO instruments. The important contribution of interpretation aids developed utilizing the IRT is that it informs clinicians and patients what performance they can expect based on a score on a multi-item instrument.

#### The MID

Irrespective of the strategy used to estimate the MID, from the individual patient point of view it is relevant to present the clinical trial results as the proportion of patients achieving a particular benefit (e.g. a MID, or any other value for that matter, be it a small, moderate, or large difference), instead of reporting only a mean difference. To calculate the proportion who achieved a MID, one must consider not only the difference between groups in those who achieve that improvement but also the difference between groups in those who deteriorate by the same amount. These differences can also be transformed into a number needed to treat required to achieve an MID in one patient after a given time period.

## Conclusion

Estimation of an MID and interpretation of clinical trial results that present patient important outcomes is as demanding as it is vital in informing the decision to recommend or not to recommend or approve a given intervention. Investigators should be encouraged to use reliable and valid methods to achieve meaningfulness of their results, preferably those that rely on patients to estimate the MID. Ideally, the different approaches to estimating the MID will produce similar results. If they do not, this should be explicitly labelled. The FDA will have to provide more specific guidance than what is offered in the current document as to which methods and approaches are preferred. Clinical investigators will benefit from such advice, since it will let them avoid designing or selecting approaches that are likely not to be valid and, therefore, not accepted by the regulators. We hope that patient-based approaches will prevail as the perspective of the patients or their informed proxies for conditions that render patient decisions difficult (e.g. end of life decisions). At a minimum all approaches should be patient-driven and involve scenarios and vignettes, but not solely a clinician's judgment. We agree with the authors of the parallel comment that demonstrating responsiveness is a key component of demonstrating appropriate measurement properties an instrument [57]. We believe the MID of a generic instrument, however, should not vary by population and context because it questions the use of the PRO measure as a generic instrument [9]. In regards to reporting of PRO measures it is advisable that investigators report the proportion of patients achieving that benefit.

## Competing interests

HJS and GHG are authors of the CRQ. McMaster University and a research account used by HJS and GHG receive licensing fees from the use of the CRQ. There are no other competing interests related to this work.

## Authors' contributions

JB and HJS developed an outline of this article based on many discussions with GG. JB wrote the first draft of the article and HJS and GG critically revised it.

## References

[B1] Federal Drug Administration Guidance for Industry. Patient-Reported Outcome Measures: Use in Medical Product Development to Support Labeling Claims. http://www.fda.gov/cder/guidance/5460dft.pdf.

[B2] Feinstein AR (1999). Indexes of contrast and quantitative significance for comparisons of two groups. Stat Med.

[B3] Naylor CD, Llewellyn-Thomas HA (1994). Can there be a more patient-centred approach to determining clinically important effect sizes for randomized treatment trials?. J Clin Epidemiol.

[B4] Bobbio M, Demichelis B, Giustetto G (1994). Completeness of reporting trial results: effect on physicians' willingness to prescribe. Lancet.

[B5] Hux JE, Levinton CM, Naylor CD (1994). Prescribing propensity: influence of life-expectancy gains and drug costs. J Gen Intern Med.

[B6] Naylor CD, Chen E, Strauss B (1992). Measured enthusiasm: does the method of reporting trial results alter perceptions of therapeutic effectiveness?. Ann Int Med.

[B7] Redelmeier DA, Tversky A (1990). Discrepancy between medical decisions for individual patients and for groups. New Engl J Med.

[B8] Guyatt GH, Feeny DH, Patrick DL (1993). Measuring health-related quality of life. Ann Int Med.

[B9] Schünemann HJ, Guyatt GH (2005). Commentary – goodbye M(C)ID! Hello MID, where do you come from?. Health Serv Res.

[B10] Schünemann HJ, Puhan M, Goldstein R, Jaeschke R, Guyatt GH (2005). Measurement Properties and Interpretability of the Chronic Respiratory Disease Questionnaire (CRQ). J COPD.

[B11] Puhan MA, Behnke M, Devereaux PJ, Montori VM, Braendli O, Frey M, Schünemann HJ (2004). Measurement of agreement on health-related quality of life changes in response to respiratory rehabilitation by patients and physicians – a prospective study. Respir Med.

[B12] Sneeuw KC, Sprangers MA, Aaronson NK (2002). The role of health care providers and significant others in evaluating the quality of life of patients with chronic disease. J Clin Epidemiol.

[B13] Ubel PA, Loewenstein G, Jepson C (2003). Whose quality of life? A commentary exploring discrepancies between health state evaluations of patients and the general public. Qual Life Res.

[B14] von Essen L (2004). Proxy ratings of patient quality of life – factors related to patient-proxy agreement. Acta oncologica (Stockholm, Sweden).

[B15] Devereaux PJ, Anderson DR, Gardner MJ, Putnam W, Flowerdew GJ, Brownell BF, Nagpal S, Cox JL (2001). Differences between perspectives of physicians and patients on anticoagulation in patients with atrial fibrillation: observational study. BMJ.

[B16] Santanello NC, Zhang J, Seidenberg B, Reiss TF, Barber BL (1999). What are minimal important changes for asthma measures in a clinical trial?. Eur Respir J.

[B17] Guyatt GH, Osoba D, Wu AW, Wyrwich KW, Norman GR (2002). Methods to explain the clinical significance of health status measures. Mayo Clin Proc.

[B18] Lydick E, Epstein RS (1993). Interpretation of quality of life changes. Qual Life Res.

[B19] Samsa G, Edelman D, Rothman ML, Williams GR, Lipscomb J, Matchar D (1999). Determining clinically important differences in health status measures: a general approach with illustration to the Health Utilities Index Mark II. PharmacoEcon.

[B20] Crosby RD, Kolotkin RL, Williams GR (2003). Defining clinically meaningful change in health-related quality of life. J Clin Epidemiol.

[B21] Deyo RA, Inui TS (1984). Toward clinical applications of health status measures: sensitivity of scales to clinically important changes. Health Serv Res.

[B22] Jaeschke R, Singer J, Guyatt GH (1989). Measurement of health status. Ascertaining the minimal clinically important difference. Contr Clin Trials.

[B23] Juniper EF, Guyatt GH, Willan A, Griffith LE (1994). Determining a minimal important change in a disease-specific Quality of Life Questionnaire. J Clin Epidemiol.

[B24] Stucki G, Liang MH, Fossel AH, Katz JN (1995). Relative responsiveness of condition-specific and generic health status measures in degenerative lumbar spinal stenosis. J Clin Epidemiol.

[B25] Thompson MS, Read JL, Hutchings HC, Paterson M, Harris ED (1988). The cost effectiveness of auranofin: results of a randomized clinical trial. J Rheumatol.

[B26] Ware JE, Keller SD, Spilker B (1996). Interpreting general health measures. Quality of Life and Pharmacoeconomics in Clinical Trials.

[B27] Brooks WB, Jordan JS, Divine GW, Smith KS, Neelon FA (1990). The impact of psychologic factors on measurement of functional status. Assessment of the sickness impact profile. Med Care.

[B28] Deyo RA, Inui TS, Leininger JD, Overman SS (1983). Measuring functional outcomes in chronic disease: a comparison of traditional scales and a self-administered health status questionnaire in patients with rheumatoid arthritis. Med Care.

[B29] Fletcher A, McLoone P, Bulpitt C (1988). Quality of life on angina therapy: a randomised controlled trial of transdermal glyceryl trinitrate against placebo. Lancet.

[B30] McSweeny AJ, Grant I, Heaton RK, Adams KM, Timms RM (1982). Life quality of patients with chronic obstructive pulmonary disease. Arch Intern Med.

[B31] King MT (1996). The interpretation of scores from the EORTC quality of life questionnaire QLQ-C30. Qual Life Res.

[B32] Bergner M, Bobbitt RA, Carter WB, Gilson BS (1981). The Sickness Impact Profile: development and final revision of a health status measure. Med Care.

[B33] Mossey JM, Shapiro E (1982). Self-rated health: a predictor of mortality among the elderly. Am J Pub Health.

[B34] Idler EL, Angel RJ (1990). Self-rated health and mortality in the NHANES-I Epidemiologic Follow-up Study. Am J Pub Health.

[B35] Brook RH, Ware JE, Rogers WH, Keeler EB, Davies AR, Donald CA, Goldberg GA, Lohr KN, Masthay PC, Newhouse JP (1983). Does free care improve adults' health? Results from a randomized controlled trial. New Engl J Med.

[B36] Fayers PM, Machin D (2000). Quality of life: assessment, analysis and interpretation.

[B37] Ware JE, Manning WG, Duan N, Wells KB, Newhouse JP (1984). Health status and the use of outpatient mental health services. Am Psychol.

[B38] Brant R, Sutherland L, Hilsden R (1999). Examining the minimum important difference. Stat Med.

[B39] Deyo RA, Centor RM (1986). Assessing the responsiveness of functional scales to clinical change: an analogy to diagnostic test performance. J Chron Dis.

[B40] Stratford PW, Binkley JM, Riddle DL, Guyatt GH (1998). Sensitivity to change of the Roland-Morris Back Pain Questionnaire: part 1. Phys Ther.

[B41] Ward MM, Marx AS, Barry NN (2000). Identification of clinically important changes in health status using receiver operating characteristic curves. J Clin Epidemiol.

[B42] Husted JA, Cook RJ, Farewell VT, Gladman DD (2000). Methods for assessing responsiveness: a critical review and recommendations. J Clin Epidemiol.

[B43] Speer DC, Greenbaum PE (1995). Five methods for computing significant individual client change and improvement rates: support for an individual growth curve approach. J Consul Clin Psychol.

[B44] Cohen J (1988). Statistical Power Analysis for the Behavioral Sciences.

[B45] Kazis LE, Anderson JJ, Meenan RF (1989). Effect sizes for interpreting changes in health status. Med Care.

[B46] Guyatt GH, Bombardier C, Tugwell PX (1986). Measuring disease-specific quality of life in clinical trials. CMAJ.

[B47] Wyrwich KW, Tierney WM, Wolinsky FD (1999). Further evidence supporting an SEM-based criterion for identifying meaningful intra-individual changes in health-related quality of life. J Clin Epidemiol.

[B48] Jacobson NS, Truax P (1991). Clinical significance: a statistical approach to defining meaningful change in psychotherapy research. J Consul Clin Psychol.

[B49] Redelmeier DA, Guyatt GH, Goldstein RS (1996). Assessing the minimal important difference in symptoms: a comparison of two techniques. J Clin Epidemiol.

[B50] Osoba D, Rodrigues G, Myles J, Zee B, Pater J (1998). Interpreting the significance of changes in health-related quality-of-life scores. J Clin Oncol.

[B51] Best WR, Becktel JM, Pena AS, Weterman IT, Booth C, Strober W (1981). The Crohn's disease activity index as a clinical instrument. Developments in Gastroenterology: Recent Advances in Crohn's Disease.

[B52] Redelmeier DA, Guyatt GH, Goldstein RS (1996). On the debate over methods for estimating the clinically important difference. J Clin Epidemiol.

[B53] Schünemann HJ, Akl EA, Guyatt GH (2006). Interpreting the Results of Patient Reported Outcome Measures in Clinical Trials: The Clinician's Perspective. Health Qual Life Outcomes.

[B54] Guyatt GH, Juniper EF, Walter SD, Griffith LE, Goldstein RS (1998). Interpreting treatment effects in randomised trials. BMJ Clinical research ed.

[B55] Stewart AL, Greenfield S, Hays RD, Wells K, Rogers WH, Berry SD, McGlynn EA, Ware JE (1989). Functional status and well-being of patients with chronic conditions. Results from the Medical Outcomes Study. JAMA.

[B56] Valderas JM, Alonso J, Prieto L, Espallargues M, Castells X (2004). Content-based interpretation aids for health-related quality of life measures in clinical practice. An example for the visual function index (VF-14). Qual Life Res.

[B57] Revicki DA, Cella D, Hays RD, Sloan JA, Lenderking WR, Aaronson NK (2006). Responsiveness and minimal important differences for patient reported outcomes. Health Qual Life Outcomes.

